# Steroid cell tumor: a rare cause of hirsutism in a female

**DOI:** 10.1530/EDM-13-0030

**Published:** 2013-09-16

**Authors:** Jayshree Swain, Shruti Sharma, Ved Prakash, N K Agrawal, S K Singh

**Affiliations:** Department of EndocrinologyInstitute of Medical Sciences, Benaras Hindu UniversityVaranasi, 221005India

## Abstract

Ovarian steroid cell tumors are very rare functioning sex-cord stromal tumors. They comprise <0.1% of all ovarian tumors. Previously designated as lipoid cell tumors, one-third of these tumors are considered malignant with the mean age of presentation at around 40 years. We present a case of a 28-year-old female with 2-year history of hirsutism, virilization, and amenorrhea. She was diagnosed with left ovarian tumor, for which she underwent left salpingo-oophorectomy. Histopathology revealed not otherwise specified subtype of steroid cell tumors. The patient resumed menses 2 months after the features of masculinization regressed. Within 1 year of surgery, the patient successfully conceived a full-term baby without any complications. In a young female, the neoplastic etiology of a rapid virilization or menses changing should always be kept in mind. Though commonly observed in adult females, steroid cell tumors have very good surgical outcomes if age at presentation is less and tumor is unilateral, and there are no evidences of bilateral malignancy. Bilateral salpingo-oophorectomy is not required.

**Learning points:**

- In a case of severe rapid hirsutism and virilization with serum testosterone level more than 200 ng/dl or more than threefold of the normal range, neoplastic conditions should always be suspected.
- Steroid cell tumor in young women without evidence of malignancy on histopathology has excellent surgical outcomes.
- Unilateral salpingo-oophorectomy is the surgery of choice.
- As the frequency of bilateralism is only 6%, prophylactic unaffected side oophorectomy need not be done.

## Background

Ovarian steroid cell tumors are very rare functioning sex-cord stromal tumors. They comprise <0.1% of all ovarian tumors (1). A subtype of this tumor called not otherwise specified (NOS) accounts for approximately one-half of all the steroid cell tumors. Approximately one-third of steroid cell tumors in adults have been reported to be malignant. Previously, these tumors were referred to as lipid or lipoid cell tumors of ovary.

Herein, we present a case of a 28-year-old female with NOS subtype of steroid cell tumor, who presented with hirsutism, virilization, and amenorrhea and a had successful response to surgery during follow-up.

## Case report

**Figure 1:**
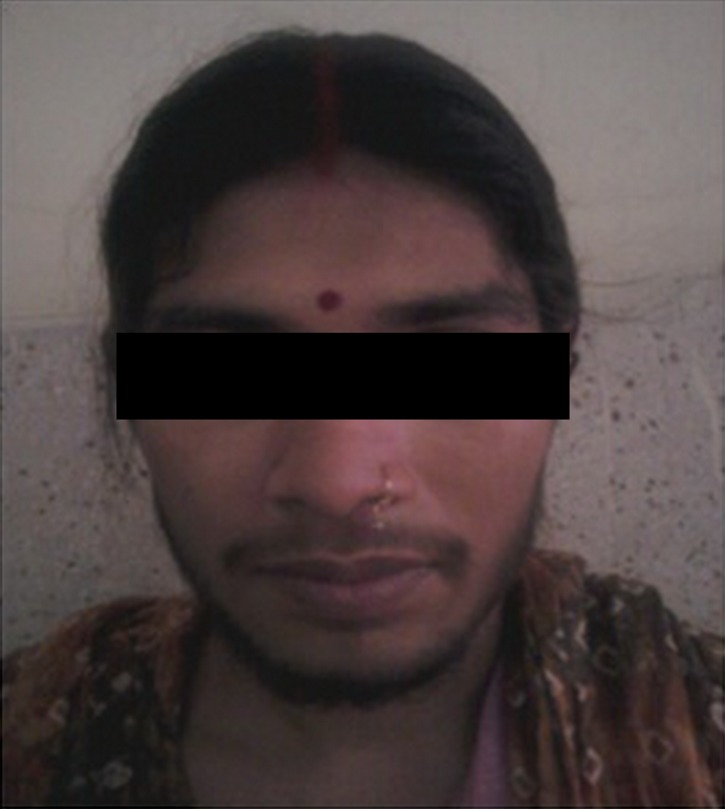
A 28-year-old female with severe virilization.

A 28-year-old female (Fig. 1) presented to the Endocrinology unit with a 2-year history of hirsutism, virilization, and amenorrhea. Her last childbirth was 2 years back with lactation failure. Previously, she had regular menstrual cycles. There was no history of gestational hirsutism, no history of drug intake, headache, proximal muscle weakness, striae or galactorrhea. The patient reported a history of mild intermittent pain in the abdomen.

On physical examination, it was found that she had male body habitus with normal vital signs. She also had: temporal recession of hairline; acne (grade 2); prominent Adam's apple; and atrophy of bilateral mammary gland. Her Ferriman–Gallwey score was 30/36. Clitoromegaly with a length of 90 mm and a width of 30 mm was present, with a clitoral index of 2700 mm^2^. Systemic examination revealed no abnormalities.

## Investigation

Laboratory findings included normal blood cell count, electrolyte level, renal, and hepatic profile. Additional measures were: serum total testosterone level, 699.32 ng/dl (normal <60 ng/dl); DHEA-S, 0.8 μg/ml (normal <2 μg/ml); serum thyrotropin, prolactin, and cortisol values were 2.5 μIU/ml (normal 0.5–4 μIU/ml), 8 ng/ml (normal 2–20 ng/ml), and 16 μg/l (normal 5–25 μg/l), respectively; basal 17 hydroxyprogesterone, 0.6 ng/ml (<3 ng/ml); follicle-stimulating hormone and luteinizing hormone values were 6 and 4 IU/l; and tumor markers Ca19-9, Ca15-3, carcinoembryonic antigen (CEA), and alpha-fetoprotein were within normal limits.

Ultrasonography of the abdomen revealed left ovarian tumor, which was confirmed by contrast-enhanced computed tomography (CECT) of the abdomen showing a well-defined lesion, which measured 5.2×5.9×5.9 cm. The lesion was lobulated, highly enhancing, with a density similar to soft tissue, and had nonenhancing necrotic areas. Ascites was not present.

## Treatment

**Figure 2:**
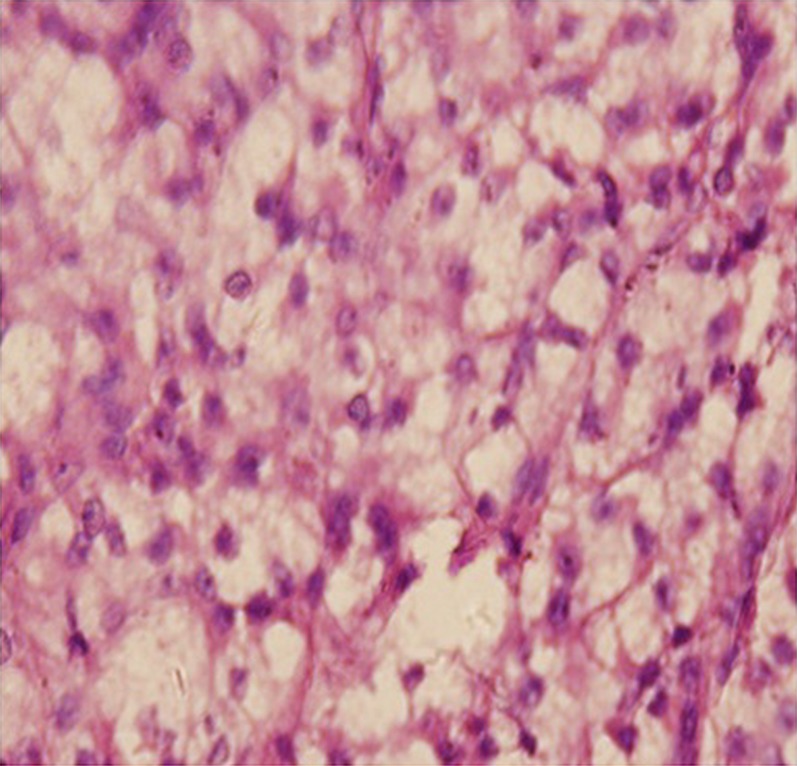
Histopathology of the tumor showing vacuolated clear cells suggestive of NOS subtype of steroid cell tumor.

The patient underwent left salpingo-oophorectomy. Histopathology revealed granular to eosinophilic tumor cells with a clear appearance, with a moderate amount of cytoplasm. Some of these cells had a vacuolated clear appearance suggestive of NOS subtype of steroid cell tumor (Fig. 2).

## Outcome and follow-up

During the first month of the follow-up, the patient's serum testosterone was 22 ng/dl.

She resumed her menstrual cycles within 2 months of the operation with regression of masculinizing signs. Two months after the resumption of menses, she conceived and gave birth to a full-term healthy baby.

## Discussion

In a case of rapid-onset hirsutism, virilization, and menstrual irregularity, neoplastic etiology should always be kept in mind. Therefore, comprehensive workup is required. Serum testosterone and DHEA-S assays are the first tests used for the evaluation of either an adrenal or ovarian source of pathology for the hyperandrogenism. Serum testosterone level above 200 ng/dl is the important diagnostic threshold level for the discrimination of neoplastic source from other nonneoplastic causes of hirsutism. Adrenal tumors causing virilization are very rare. They can be either adenomas with a size of 1–2 cm or carcinomas with a size >4 cm and secrete other hormones in addition to testosterone, usually glucocorticoids and sometimes estrogens. The majority of virilising tumors are thus ovarian in origin and include arrhenoblastomas (Sertoli–Leydig cell tumors), hilus cell tumors, steroid cell tumors, and infrequently, granulosa–theca tumor. Nonfunctional ovarian neoplasms such as epithelial cystadenomas or cystadenocarcinomas may rarely present with androgen excess by stimulating steroidogenesis in the adjacent nonneoplastic ovarian stroma. Our patient's testosterone level was 699 ng/dl with normal DHEA-S. Prior case reports of ovarian steroid cell tumor (NOS subtype) were misdiagnosed with late-onset congenital adrenal hyperplasia. Therefore, serum basal 17 (OH) progesterone was assayed in our patient and was found to be normal (2). The next step was to identify the tumor with abdominal ultrasound, CT scan, or MRI. Both pelvic ultrasound and CECT of the abdomen in our patient confirmed the presence of unilateral solid ovarian tumor without local invasion or metastasis. The tumor was surgically resected and histologically confirmed as NOS steroid cell tumor.

The ovarian steroid cell tumor was first described by Scully, who reported 63 cases ranging from 2 to 80 years of age (3). Previously, it was known as lipid or lipoid cell tumor, though sometimes it has little or no lipid content. Steroid cell tumors derive from adrenal rest cells, ovarian stromal lutein cells, or Leydig cells.

Steroid cell tumors have been classified into three subtypes – NOS, Leydig cell tumor, and stromal luteoma (4). NOS subtype includes two types of polygonal cells that differ only in their cytoplasmic appearance (eosinophilic vs vacuolated). In a series of cases from Massachusetts General Hospital, 94% of the tumors were found to be unilateral and 28.6% were malignant (5).

Around 56% of patients present with hirsutism and virilization, though estradiol secretion is detectable in 6–23% of these patients (6). Other clinical symptoms include menorrhagia and irregular bleeding after menopause. Cushing's syndrome has also been reported (4).

The first-line treatment is surgery. In older women, total abdominal hysterectomy and bilateral salpingo-oophrectomy are the appropriate management options, while in young women, unilateral salpingo-oophrectomy is adequate in most cases if histology shows no malignant features. Regular follow-up evaluation with measurement of serum testosterone level is mandatory (7).

Additionally a gonadotropin-releasing hormone agonist could be used as postoperative adjuvant therapy. Patients with large ovarian tumors and with an advanced stage have worse prognosis.

Malignant NOS steroid cell tumors should be managed with surgical removal followed by a combination of chemotherapy and radiotherapy (7). Most of these tumors are diagnosed at an early stage due to signs and symptoms and do not recur and metastasize. So, the therapeutic value of chemotherapy and radiotherapy is controversial (7).

This case is unique because of its presentation in young age, with the sudden onset of amenorrhea during lactation and signs of virilization over a short period, and because of the resumption of menses and successful pregnancy within one year of surgery.

## Patient consent

Written informed consent was obtained from the patient for publication of this case report.

## Author contribution statement

Dr J Swain and Dr S Sharma drafted the first manuscript for submission, which was conceptualized by Dr N K Agrawal and Dr V Prakash. The final draft of the manuscript was revised by Prof. S K Singh and he gave final consent for manuscript submission. The manuscript was revised by Prof. S K Singh accordingly. Dr J Swain and Prof. S K Singh were the primary physicians of the patient and the patient was in their follow-up.
